# Inflammatory Markers and Risk of Parkinson's Disease: A Population-Based Analysis

**DOI:** 10.1155/padi/4192853

**Published:** 2024-12-31

**Authors:** Hongping Wang, Wenqiang Li, Qun Lai, Qian Huang, Hao Ding, Zhiping Deng

**Affiliations:** ^1^Department of Neurosurgery, Zigong Fourth People's Hospital, 19 Tanmulin Street, Zigong, Sichuan, China; ^2^Department of Pulmonary and Critical Care Medicine, Zigong First People's Hospital, 42 Shangyihao Yizhi Street, Zigong, Sichuan, China; ^3^Department of Hematology, Rheumatology and Immunology, Zigong Fourth People's Hospital, 19 Tanmulin Street, Zigong, Sichuan, China; ^4^Department of Pulmonary and Critical Care Medicine, Dazhou Third People's Hospital, Dazhou, Sichuan, China

**Keywords:** lymphocyte-monocyte ratio (LMR), neutrophil-lymphocyte ratio, NHANES, Parkinson's disease (PD), platelet-lymphocyte ratio (PLR)

## Abstract

**Objected:** Parkinson's disease (PD) is an important cause of neurological dysfunction, and the aim of this study was to explore whether neutrophil-to-lymphocyte ratio (NLR), platelet-to-lymphocyte ratio (PLR), lymphocyte-to-monocyte ratio (LMR), systemic inflammatory response (SIRI), and systemic immune inflammation (SII) are associated with the risk of developing PD. Based on this, we may identify people at high risk for PD and intervene early.

**Method:** Our study included 31,480 participants from the National Health and Nutrition Examination Survey (NHANES) between 2001 and 2018. Basic information and inflammation-related indicators were obtained by questionnaires and laboratory tests, respectively. NLR, PLR, LMR, SIRI, SII, and PD risk were analyzed using weighted logistic regression models.

**Results:** There were 261 and 31,219 in the PD and non-PD groups, respectively, and the prevalence of PD was 0.83%. Separate analyses of NLR and PLR were conducted after fully adjusting for confounding factors. According to our analysis, there was an increased risk of PD for both NLR and PLR in the higher level group (Q4) as compared with the lower level group (Q1) (OR = 1.83 and 95% confidence interval (CI) = 1.09–3.07, and OR = 1.92 and 95% CI = 1.20–3.08). However, we did not find similar relationships in LMR, SIRI, and SII.

**Conclusions:** There was a significant association between elevated levels of NLR, PLR, and PD risk, while LMR, SIRI, and SII were not statistically significant. It suggests that NLR or PLR could be used to screen people at risk of PD at an early stage. It is essential to conduct more large-scale prospective studies to investigate the role that NLR and PLR play in PD.

## 1. Introduction

Parkinson's disease (PD) is a neurodegenerative disease that is a significant cause of neurological dysfunction, with a prevalence rate of 4% [1]. Symptoms of PD include impairments in motor (muscle stiffness, resting tremor, and postural reflex deficits) and nonmotor abilities (sleep disturbances, olfactory abnormalities, depression/anxiety, and cognitive deficits) [2]. These pathologies are associated with cell loss in the *α*-synaptic nuclear protein and regions composed of syn in the Lewy bodies and neurons in the substantia nigra [3]. Typically, nonmotor symptoms (NMSs) appear gradually as PD evolves. Interestingly, some studies suggested that NMS may manifest before motor symptoms, and the likelihood that dementia occurs in later stages of the disease is as high as 80% [4, 5]. This high probability may be due to neuroinflammation that affects various systems, including the dopamine and cholinergic systems [6]. PD not only significantly impacts the quality of life of patients but also imposes a considerable socioeconomic burden. Despite recent advances in therapies, such as new medications, alternative routes of administration, and surgical interventions, the mental and psychological challenges that accompany this disease in its later stages are substantial. Therefore, a better understanding of potential risk factors for PD and implementing preventive measures are crucial steps in addressing this complex condition.

Research has shown that genetic factors, age, sex, environmental factors, and viral/bacterial infections are important risk factors for PD [7, 8]. Approximately 5%–10% of PD cases are genetically predisposed and may be associated with genetic mutations [9, 10]. Patients with confirmed PD among their family members have a risk of developing PD more than four times higher than individuals without such a family history [11, 12]. Age is an independent risk factor for both the occurrence and severity of PD, with its incidence rate increasing with age [7]. Gender also plays an important role, with a prevalence rate of approximately 3:2 between men and women. Furthermore, studies have indicated a positive correlation between pesticides, heavy metals, and the incidence rate of PD [13]. It has been suggested that the neuroinflammation resulting from the interaction of central and peripheral immune cells has a significant impact on the development of PD [2, 14–16]. There have been previous studies that have shown that changes in neutrophils and lymphocytes occur years before PD is diagnosed [17, 18]. PD risk was higher among individuals with lower lymphocyte counts in a large UK cohort [17]. A comparison of PD patients with healthy individuals revealed that the neutrophil count in PD patients was higher and lymphocyte count was lower [19–21]. During pre-PD or early PD, peripheral blood mononuclear cells increase in activation, possibly as a result of their early involvement in innate immunity [22]. There is strong evidence that inflammation plays a critical role in the development of PD. However, up to now, there is still a lack of research on the relationship between the risk of developing PD and overall systemic inflammation and immune status. Neutrophil-to-lymphocyte ratio (NLR), platelet-to-lymphocyte ratio (PLR), lymphocyte-to-monocyte ratio (LMR), systemic inflammatory response (SIRI), and systemic immune inflammation (SII) have been applied as novel biomarkers of inflammation to evaluate the association between chronic inflammation and cancer, stroke, and metabolic disorders [23–26]. However, the correlation between these combined inflammatory markers and PD is unclear.

Therefore, utilizing data from a large sample of the National Health and Nutrition Examination Survey (NHANES), the current study explored, for the first time, the correlation between NLR, PLR, LMR, SIRI, SII, and with the risk of developing PD. With this screening, it is hoped that people at risk for PD will be identified, and that early diagnosis and intervention in patients with PD will improve their prognosis.

## 2. Methods

### 2.1. Study Population

NHANES is a complex, multistage study of adults and children in the U.S. It consists of two important parts: (1) an interview conducted face-to-face by a professional through a specialized questionnaire or a self-assessment by the participant through a self-administered questionnaire and (2) the other part is obtained by laboratory tests such as blood, glucose, and blood pressure (https://www.cdc.gov/nchs/nhanes/index).

**Figure 1:**
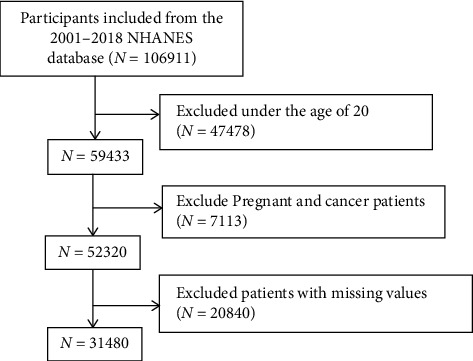
Flowchart of participant selection (NHANES, National Health and Nutrition Examination Survey).

There were 106,911 participants in the initial survey. To ensure age consistency, we excluded 47,478 participants under the age of 20. Also, pregnant women and cancer patients themselves have changes in inflammatory cell counts that may interfere with the results of the study [27, 28], so 1350 pregnant women and 5763 cancer patients. In addition, study participants with a history of PD or missing data on relevant covariates were excluded (*N* = 20,840), resulting in a final sample of 31,480 participants **(**Figure 1**)**.

### 2.2. Diagnosis of PD

In the NHANES database, in order to determine whether a participant had PD, it was dependent on the participant's reported use of prescription medications (defined as carbidopa, levodopa, methyldopa, phenytoin, ropinirole, entacapone, and amantadine) that are specifically designed to treat PD. When participants were treated with “anti-PD medications,” they were classified as having PD, whereas other participants were not. Although there may be patients who are missed in this way, based on the NHANES study of PD patients, this diagnostic criterion is still scientifically valid and has been widely adopted in many studies [29, 30].

### 2.3. Exposure Factors

The counts of lymphocytes, monocytes, neutrophils, and platelets were obtained using an automated hematology analysis device (Coulter DxH 800 analyzer) and present as × 103 cells/μL. As part of the NHANES Laboratory Manual, standardized protocols for measuring the biomarkers listed above are provided, as well as an explanation of any biases that may exist (https://www.cdc.gov/nchs/nhanes/biospecimens/serum_plasma_urine.htm). Five markers of systemic inflammation were calculated: PLR, NLR, LMR, SIRI, and SII. The formula is as follows: NLR = neutrophil counts/lymphocyte counts, PLR = platelet counts/lymphocyte counts, LMR = lymphocyte counts/monocyte counts, SIRI = neutrophil counts⁣^∗^ monocytes counts/lymphocytes counts, SII = platelet counts⁣^∗^ neutrophil counts/lymphocyte counts [25, 26, 31].

### 2.4. Covariate Information

In our study, some key covariates were considered. These include basic information, socioeconomic status, lifestyle habits, and underlying diseases. A number of demographic factors were measured, including age, gender, race, and body mass index (BMI, categorized as ≤ 25, 25–30, and ≥ 30). Life factors include (1) family poverty to income ratio (PIR, divided into ≤ 1, 1–3, and ≥ 3); (2) education level; (3) smoking was divided into past smoking (smoking > 100 cigarettes/lifetime, now successfully quit smoking), current smoking (smoking > 100 cigarettes/lifetime), and never smoking (smoking < 100 cigarettes/lifetime); (4) alcohol consumption was categorized as drinking and nondrinking; (5) Healthy Eating Index 2015 (HEI-2015) is an indicator for evaluating dietary quality, higher scores indicate better dietary quality [32]; and (6) energy (kcal) intake was likewise included as a covariate in the analysis. We collected the abovementioned covariates using standardized questionnaires and test volumes.

Clinical underlying diseases include (1) hypertension, which is diagnosed by a professional through a questionnaire, a history of oral hypertension medication, and laboratory tests. (2) Diabetes, diagnosed by professionals in the questionnaire/with oral antihypertensive drugs/history of insulin use/laboratory indicators, meets the diagnostic criteria for diabetes. (3) History of chronic cardiovascular disease (CVD), including diagnosis of coronary heart disease, chronic heart failure, heart attack, stroke, and angina by relevant medical professionals.

### 2.5. Statistical Analysis

R 4.2.2 was used for data analysis in this study. Percentages were used for categorical variables. Comparisons between the two groups were performed using the *χ*^2^ tests (categorical variables) and Kruskal–Wallis tests (skewed distribution). Multivariate logistic regression models were used to determine whether NLR, PLR, MLR, SIRI, and SII were related to PD. Odds ratio (OR) and 95% confidence interval (CI) were calculated after adjusting for confounding factors. There were no covariates adjusted in the coarse model. Model 1 has made adjustments to sex, age, race, marriage, and education. Model 2 further adjusted smoking, PIR, insurance, and drinking. Based on Model 2, Model 3 was further adjusted for BMI, diabetes, hypertension, HEI-2015, energy, and cardiovascular disease. To avoid oversampling and no response, sample weights were used for data analysis in this study.

## 3. Results

### 3.1. Baseline Characteristics of the Participants

There were 31,480 participants in total, of which 261 had PD (0.83% prevalence), out of 15,864 male and 15,616 female participants **(**Table 1**)**. In regards to age, marital status, PIR, insurance, diabetes, hypertension, and CVD, there was a significant difference between the two groups (*p* < 0.05). In the PD group, 77.12% of the patients were older than 45 years of age and 53.98% were hypertensive. The differences between the two groups were not significant with regards to gender, race, education, BMI, smoking, drinking, and HEI-2015 (*p* ≥ 0.05).

### 3.2. Relationship Between NLR, PLR, LMR, SIRI, SII, and PD

A weighted logistic regression model was used to investigate the relationship between NLR, PLR, LMR, SIRI, SII, and PD risk (Table 2).

Adjusted analyses showed that the highest NLR quartile (Q4) was significantly associated with an increased risk of PD compared with the lowest quartile (Q1) (OR = 1.83, 95% CI = 1.09, 3.07, and *p* < 0.05). PLR in Q4 was significantly associated with increased risk of PD compared with Q1 (OR = 1.92, 95% CI = 1.20, 3.08, and *p* < 0.05). As for LMR, SIRI, and SII, they did not appear to be significantly related to the risk of developing PD (Table 2).

### 3.3. Subgroup Analysis

A subgroup analysis was also conducted to examine the correlation between NLR and PD in more detail. There was no statistically significant difference in the correlation between NLR and PD between different confounders, indicating that age, gender, marital status, race, education, PIR, and BMI did not have a significant effect on this positive correlation (*p*_interaction_ > 0.05) (Table 3).

Furthermore, we conducted a subgroup analysis of the correlation between PLR and PD and found no effect from subgroup analysis on the positive correlation (*p*_interaction_ > 0.05) (Table 4).

## 4. Discussion

As far as we know, this is the first large-sample study to examine the association between multiple inflammatory markers and PD. The results of an analysis of 31,480 adults found that NLR and PLR exhibited positive associations with the risk of developing PD, whereas LMR, SIRI, and SII showed no significant association with the risk. There were no differences in these associations based on gender, age, race, marriage, education, PIR, and BMI. A full adjustment for confounders showed that PD prevalences were 1.83 and 1.92 times higher in Q4 participants with NLR and PLR than in Q1. The findings suggest that NLR and PLR may be useful in the future for predicting the risk of developing PD.

Aging is an independent risk factor for the onset and progression of PD [7, 13]. The incidence and prevalence of PD has also increased in recent years with aging [33]. According to existing studies, about 572/100,000 people aged 45 years and older have PD [34]. In this study, the prevalence of PD in individuals aged 45 and older was about 211 out of 17,256, aligning with previous findings and reinforcing this conclusion. The body's immune system and inflammation change with age.

There is a connection between PD's pathogenesis and inflammation-mediated oxidative stress, mitochondrial dysfunction, and cytokine toxicity-induced neuronal damage related to chronic inflammation [35–37]. Inflammation is the body's protective mechanism against pathogenic stimuli or tissue damage and promotes tissue self-repair. Blood-brain barriers (BBBs) protect the central nervous system, one of the body's most important organs. An inflammatory condition alters the BBB's permeability, which increases immune cell infiltration into the brain [38, 39]. Activation of CD4+ *T* cells stimulates microglia and *α*-synuclein, a protein that triggers both innate and adaptive immune responses, leading to sustained neuroinflammation [40–42]. In the early stages of the immune system, monocytes play a central role. With the activation of the innate and adaptive (acquired) immune system, TLR 4 expression in monocytes correlates directly with immune brain activation and indirectly with dopaminergic neurotransmission [22, 43]. It is also important to note that monocyte-derived macrophages and dendritic cells play an important role in orchestrating inflammation and reversing it [44, 45]. Lymphocytes play a crucial role in the adaptive immune system. There is a reduction of lymphocytes in the blood of patients before the diagnosis of PD, which links innate and adaptive immune responses, influencing the disease's development [17, 18]. The development of PD is linked to oxidative stress. Increased reactive oxygen species in the platelets of the peripheral blood of PD patients alters platelet activity and assists in creating an inflammatory environment [46, 47].

There has been evidence that PD is associated with changes in neutrophil, monocyte, lymphocyte, and platelet counts and activity [15, 17–22]. A case-control study, which found increased neutrophil counts and decreased lymphocyte counts in those diagnosed with PD compared with the healthy population, led to the conclusion that NLR was higher in patients with PD [48]. Our findings that there is a high risk of PD associated with NLR at Q4 are consistent with this finding. In other words, PD may develop when the protective immune function of lymphocytes is significantly weakened, in conjunction with chronic inflammation caused by increased neutrophils [49–51]. Platelets serve as a key inflammatory and immune marker in peripheral blood. This study identified that individuals in the Q4 quartile of the PLR had a 1.92-fold increased risk of PD compared with those in the Q1 quartile. Prior research indicates that peripheral platelet counts in patients with PD are either unchanged or reduced [52, 53]. It is evident that platelets contribute to the progression of PD in a way that is less related to platelet counts and may be due to their aberrant structural alterations (including increased size, distorted shape, and stretching) and functional changes (hyperactivation and defective adhesion, etc.) [52, 54–56]. Increased PLR may increase the risk of developing PD, possibly due to decreased lymphocyte counts. In addition, we examined the relationship between LMR, SIRI, SII, and PD. Although no significant correlation with PD was demonstrated in the current study, a large number of studies have demonstrated that LMR, SIRI, and SII are strongly associated with the development and prognosis of many chronic inflammatory diseases, including hypertension, stroke, cancer, and metabolism-related diseases [25, 26, 28, 57–59].

Interestingly, 53.09% of the PD patients did not smoke, which supports previous studies claiming that smoking protects against PD [11, 60]. Moreover, Gallo et al. [61] found that people who had previously smoked reduced their risk of PD by 20% and current smokers by 50%. Also, this potentially attributed to nicotine's impact on *α*-synuclein fibrils inhibiting free radical production [13, 62, 63]. Speculatively, nicotine can stimulate the striatum to release dopamine, influencing the production of inflammatory cytokines and cell apoptosis or other neuroprotective functions present in tobacco [11, 60, 64].

Our study boasts several notable strengths. For the first time, we utilized a large population sample to investigate the impact of a composite peripheral blood inflammation index on PD risk. In addition, the current study incorporated a large number of potential confounding variables, such as underlying sociodemographic characteristics, lifestyle factors, nutritional intake, energy intake, and clinical underlying diseases, which effectively increased the credibility and robustness of the findings. Nevertheless, this study has several limitations. First, cross-sectional studies lack dynamic follow-up data, so causality cannot be inferred. In addition, the diagnosis of PD based on self-reported use of antiparkinsonian medications may not be accurate enough, which may introduce some statistical error. Finally, although many covariates have been considered, there are still unmeasured confounders, such as genetic factors, environmental factors, and substance use. Moving forward, it is recommended that large-scale longitudinal studies be carried out to validate and elucidate the causal relationship between these two variables.

## 5. Conclusion

In summary, a significant correlation was found between elevated levels of NLR and PLR and the risk of PD. NLR and PLR may thus be a promising predictor of PD. These preliminary findings will need to be validated through future multicenter cohort studies due to the limitations of cross-sectional studies.

## Figures and Tables

**Table 1:** Characteristics of study population.

Variable	Total	Non-PD	PD	*p* value
Sample, *n* (%)	*N* = 31,480	*N* = 31,219 (99.17)	*N* = 261 (0.83)	
Sex, *n* (%)				0.05
Female	15,616 (49.71)	15,476 (50.23)	140 (58.66)	
Male	15,864 (50.29)	15,743 (49.77)	121 (41.34)	
Age, years, *n* (%)				< 0.0001
≤ 44	14,224 (49.36)	14,174 (49.56)	50 (22.88)	
45–65	10,856 (36.33)	10,756 (36.23)	100 (48.52)	
≥ 65	6400 (14.32)	6289 (14.21)	111 (28.60)	
Marriage, *n* (%)				0.01
Married/living with partner	19,084 (62.93)	18,941 (62.98)	143 (56.60)	
Never married	5775 (19.37)	5735 (19.40)	40 (15.17)	
Widowed/divorced/separated	6621 (17.70)	6543 (17.62)	78 (28.23)	
Race, *n* (%)				0.1
Black	6574 (10.89)	6536 (10.90)	38 (9.21)	
Mexican	5421 (8.41)	5391 (8.44)	30 (4.59)	
White	14,086 (68.84)	13,923 (68.78)	163 (76.38)	
Other	5399 (11.86)	5369 (11.88)	30 (9.82)	
Education, *n* (%)				0.1
Lower than high school	7647 (15.18)	7569 (15.13)	78 (21.29)	
High school diploma	7351 (24.16)	7295 (24.13)	56 (27.53)	
More than high school	16,482 (60.67)	16,355 (60.74)	127 (51.18)	
BMI, kg/m2, *n* (%)				0.29
≤ 25	9175 (30.86)	9108 (30.88)	67 (27.81)	
25–30	10,550 (32.97)	10,477 (33.00)	73 (29.10)	
≥ 30	11,755 (36.17)	11,634 (36.12)	121 (43.09)	
PIR, *n* (%)				0.01
≤ 1 (lowest income)	9477 (21.26)	9379 (21.18)	98 (31.27)	
1–3	11,981 (35.23)	11,884 (35.24)	97 (34.99)	
≥ 3 (highest income)	10,022 (43.51)	9956 (43.58)	66 (33.74)	
Insurance, *n* (%)				< 0.001
No	6953 (18.38)	6937 (18.47)	16 (6.23)	
Yes	24,527 (81.62)	24,282 (81.53)	245 (93.77)	
Smoke, *n* (%)				0.47
Former	7423 (23.53)	7358 (23.55)	65 (21.14)	
Never	17,167 (54.51)	17,035 (54.52)	132 (53.09)	
Now	6890 (21.96)	6826 (21.93)	64 (25.77)	
Drink, *n* (%)				0.25
No	4313 (10.95)	4272 (10.92)	41 (14.07)	
Yes	27,167 (89.05)	26,947 (89.08)	220 (85.93)	
Diabetes, *n* (%)				< 0.001
Borderline	625 (1.64)	616 (1.62)	9 (3.96)	
No	27,258 (90.11)	27,058 (90.19)	200 (80.59)	
Yes	3597 (8.24)	3545 (8.19)	52 (15.44)	
Hypertension, *n* (%)				< 0.0001
No	20,999 (70.64)	20,875 (70.83)	124 (46.02)	
Yes	10,481 (29.36)	10,344 (29.17)	137 (53.98)	
HEI-2015, n (%)				0.69
Q1	7870 (25.14)	7799 (25.12)	71 (28.24)	
Q2	7870 (24.91)	7810 (24.94)	60 (21.04)	
Q3	7869 (24.68)	7799 (24.66)	70 (26.72)	
Q4	7871 (25.27)	7811 (25.28)	60 (23.99)	
Energy, *n* (%)				0.18
Q1	7874 (22.26)	7793 (22.21)	81 (28.14)	
Q2	7869 (24.90)	7795 (24.89)	74 (26.41)	
Q3	7870 (26.17)	7807 (26.16)	63 (27.46)	
Q4	7867 (26.67)	7824 (26.74)	43 (17.99)	
CVD, *n* (%)				< 0.0001
No	28,459 (92.53)	28,272 (92.65)	187 (77.93)	
Yes	3021 (7.47)	2947 (7.35)	74 (22.07)	
PLR, *n* (%)				0.05
Q1	7866 (23.95)	7810 (24.01)	56 (16.02)	
Q2	7872 (25.29)	7804 (25.25)	68 (29.98)	
Q3	7896 (25.75)	7830 (25.78)	66 (21.65)	
Q4	7846 (25.02)	7775 (24.96)	71 (32.35)	
NLR, *n* (%)				0.01
Q1	7843 (22.20)	7796 (22.27)	47 (13.80)	
Q2	7855 (25.85)	7794 (25.83)	61 (27.54)	
Q3	7907 (26.41)	7844 (26.44)	63 (22.35)	
Q4	7875 (25.54)	7785 (25.45)	90 (36.30)	
LMR, *n* (%)				0.2
Q1	8561 (24.85)	8495 (24.85)	66 (24.78)	
Q2	7632 (24.58)	7580 (24.62)	52 (19.61)	
Q3	7408 (24.74)	7355 (24.76)	53 (22.20)	
Q4	7879 (25.82)	7789 (25.76)	90 (33.40)	
SIRI, *n* (%)				0.01
Q1	7873 (21.65)	7817 (21.67)	56 (18.75)	
Q2	7920 (26.25)	7875 (26.31)	45 (17.51)	
Q3	7828 (25.94)	7765 (25.94)	63 (26.10)	
Q4	7859 (26.17)	7762 (26.08)	97 (37.64)	
SII, *n* (%)				0.07
Q1	7870 (22.27)	7816 (22.31)	54 (17.26)	
Q2	7871 (25.47)	7809 (25.49)	62 (23.49)	
Q3	7869 (26.35)	7802 (26.37)	67 (24.05)	
Q4	7870 (25.91)	7792 (25.84)	78 (35.19)	

*Note:* HEI-2015: Q1: 0–40.832; Q2: 40.832–50.197; Q3: 50.197–59.991; Q4: 59.991–95.997; Energy (Kal): Q1: 0–1441.25; Q2:1441–1948.5; Q3: 1948.5–2630; Q4: 2630–13687; PLR: Q1: 2.251–94.444; Q2: 94.444–119.13; Q3: 119.13–150; Q4: 150–920; NLR: Q1: 0.009–1.444; Q2: 1.444–1.923; Q3: 1.923–2.533; Q4: 2.533–30.666; MLR: Q1: 0.033–0.2; Q2: 0.2–0.25; Q3: 0.25–0.333; Q4: 0.333–2.263; SIRI: Q1: 0.020–0.688; Q2: 0.688–1; Q3: 1–1.464; Q4: 1.464–24.6; SII: Q1: 1.528–336.506; Q2: 336.506–470.9; Q3: 470.9–657.75; Q4: 657.75–28397.275.

Abbreviations: BMI, Body Mass Index; CVD, chronic cardiovascular disease; HEI-2015, Healthy Eating Index 2015; LMR, lymphocyte-to-monocyte ratio; NLR, neutrophil-to-lymphocyte ratio; PIR, poverty income ratio; PLR, platelet-to-lymphocyte ratio; SII, systemic immune inflammation; SIRI, systemic inflammatory response index.

**Table 2:** Relationship between inflammatory markers and PD.

Inflammatory markers	Coarse model	Model 1	Model 2	Model 3
	OR (95% CI)	OR (95% CI)	OR (95% CI)	OR (95% CI)
*NLR*				
Q1	Ref	Ref	Ref	Ref
Q2	1.72 (1.05, 2.82)⁣^∗^	1.71 (1.04, 2.79)⁣^∗^	1.73 (1.06, 2.82)⁣^∗^	1.75 (1.07, 2.85)⁣^∗^
Q3	1.36 (0.78, 2.38)	1.33 (0.75, 2.35)	1.30 (0.74, 2.31)	1.27 (0.71, 2.27)
Q4	2.30 (1.40, 3.78)⁣^∗^	1.98 (1.19, 3.28)⁣^∗^	1.95 (1.17, 3.25)⁣^∗^	1.83 (1.09, 3.07)⁣^∗^
*P*_for⁣trend_	0.01	0.054	0.066	0.123

*PLR*				
Q1	Ref	Ref	Ref	Ref
Q2	1.78 (1.02, 3.09)⁣^∗^	1.75 (1.01, 3.02)	1.83 (1.07, 3.12)⁣^∗^	1.89 (1.10, 3.25)⁣^∗^
Q3	1.26 (0.80, 1.99)	1.21 (0.77, 1.92)	1.29 (0.82, 2.04)	1.35 (0.86, 2.14)
Q4	1.94 (1.22, 3.11)⁣^∗^	1.71 (1.08, 2.70)⁣^∗^	1.84 (1.17, 2.89)⁣^∗^	1.92 (1.20, 3.08)⁣^∗^
*P*_for⁣trend_	0.036	0.121	0.061	0.047

*LMR*				
Q1	Ref	Ref	Ref	Ref
Q2	0.80 (0.48, 1.34)	0.77 (0.46, 1.29)	0.79 (0.48, 1.33)	0.78 (0.46, 1.31)
Q3	0.90 (0.55, 1.48)	0.82 (0.50, 1.33)	0.84 (0.51, 1.38)	0.82 (0.49, 1.36)
Q4	1.30 (0.81, 2.08)	1.04 (0.64, 1.71)	1.06 (0.65, 1.74)	0.99 (0.61, 1.63)
*P*_for⁣trend_	0.237	0.749	0.707	0.901

*SIRI*				
Q1	Ref	Ref	Ref	Ref
Q2	0.77 (0.46, 1.30)	0.75 (0.44, 1.28)	0.75 (0.44, 1.27)	0.75 (0.44, 1.28)
Q3	1.16 (0.72, 1.88)	1.09 (0.66, 1.81)	1.08 (0.65, 1.79)	1.03 (0.63, 1.69)
Q4	1.67 (1.03, 2.70)⁣^∗^	1.45 (0.87, 2.41)	1.38 (0.82, 2.32)	1.25 (0.74, 2.10)
*P*_for⁣trend_	0.012	0.057	0.088	0.2

*SII*				
Q1	Ref	Ref	Ref	Ref
Q2	1.19 (0.69, 2.06)	1.20 (0.69, 2.07)	1.22 (0.71, 2.10)	1.22 (0.71, 2.11)
Q3	1.18 (0.73, 1.90)	1.15 (0.71, 1.86)	1.16 (0.71, 1.89)	1.15 (0.70, 1.89)
Q4	1.76 (1.06, 2.93)⁣^∗^	1.61 (0.95, 2.72)	1.61 (0.95, 2.73)	1.53 (0.90, 2.62)
*P*_for⁣trend_	0.03	0.078	0.09	0.137

*Note:* Coarse model: no covariate was adjusted. Model 1: sex, age, race, marriage, and education were adjusted. Model 2: sex, age, race, marriage, education, smoking, PIR, insurance, and drinking were adjusted. Model 3: sex, age, race, marriage, education, smoking, PIR, insurance, drinking, BMI, diabetes, hypertension, HEI-2015, energy, and CVD were adjusted.

⁣^∗^Representative *p* < 0.05.

**Table 3:** Layered analysis of the relationship between NLR and PD.

Character	Q1	Q4 OR (95% CI)	*p*	*p*_interaction_
Sex, *n* (%)				0.78
Male	Ref	2.25 (1.24, 4.08)	0.01	
Female	Ref	1.63 (0.82, 3.24)	0.16	
Age, *n* (%)				0.36
≤ 44	Ref	4.40 (1.75, 11.07)	0.002	
45–65	Ref	1.19 (0.63, 2.25)	0.58	
≥ 64	Ref	1.61 (0.66, 3.93)	0.3	
Marriage, *n* (%)				0.07
Married/living with partner	Ref	1.49 (0.65, 3.43)	0.34	
Never married	Ref	1.97 (1.07, 3.63)	0.03	
Widowed/divorced/separated	Ref	2.90 (0.74, 11.41)	0.13	
Race, *n* (%)				0.15
White	Ref	2.07 (1.23, 3.48)	0.01	
Mexican	Ref	2.20 (0.66, 7.32)	0.2	
Other	Ref	0.25 (0.04, 1.57)	0.14	
Black	Ref	2.04 (0.62, 6.70)	0.24	
Education, *n* (%)				0.07
Lower than high school	Ref	2.04 (1.02, 4.08)	0.04	
High school diploma	Ref	1.18 (0.52, 2.67)	0.68	
More than high school	Ref	5.19 (1.84, 14.64)	0.002	
PIR, *n* (%)				0.2
≤ 1 (lowest income)	Ref	1.68 (0.82, 3.47)	0.16	
1–3	Ref	3.86 (1.26, 11.88)	0.02	
≥ 3 (highest income)	Ref	1.92 (0.72, 5.12)	0.19	
BMI, *n* (%)				0.69
< 25	Ref	2.82 (1.43, 5.56)	0.003	
25–30	Ref	0.95 (0.40, 2.24)	0.9	
> 30	Ref	2.20 (0.83, 5.82)	0.11	

**Table 4:** Layered analysis of the relationship between PLR and PD.

Character	Q1	Q4 OR (95% CI)	*p*	*p*_interaction_
Sex, *n* (%)				0.62
Male	Ref	3.27 (1.58, 6.79)	0.002	
Female	Ref	1.95 (1.00, 3.83)	0.05	
Age, *n* (%)				0.43
≤ 44	Ref	1.38 (0.45, 4.21)	0.57	
45–65	Ref	3.17 (1.56, 6.41)	0.002	
≥ 64	Ref	1.96 (0.89, 4.30)	0.09	
Marriage, *n* (%)				0.21
Married/living with partner	Ref	2.37 (0.91, 6.18)	0.08	
Never married	Ref	2.07 (1.12, 3.84)	0.02	
Widowed/divorced/separated	Ref	2.19 (0.63, 7.62)	0.22	
Race, *n* (%)				0.63
White	Ref	2.56 (1.43, 4.58)	0.002	
Mexican	Ref	3.64 (0.68, 19.41)	0.13	
Other	Ref	1.02 (0.16, 6.35)	0.99	
Black	Ref	1.35 (0.41, 4.45)	0.62	
Education, *n* (%)				0.55
Lower than high school	Ref	2.81 (1.39, 5.66)	0.004	
High school diploma	Ref	1.48 (0.44, 4.93)	0.53	
More than high school	Ref	2.13 (0.83, 5.48)	0.11	
PIR, *n* (%)				0.74
≤ 1 (lowest income)	Ref	1.66 (0.75, 3.67)	0.21	
1–3	Ref	2.02 (0.82, 5.00)	0.13	
≥ 3 (highest income)	Ref	4.28 (1.51, 12.08)	0.01	
BMI, *n* (%)				0.68
< 25	Ref	1.73 (0.81, 3.66)	0.15	
25–30	Ref	2.95 (1.10, 7.92)	0.03	
> 30	Ref	2.61 (0.92, 7.41)	0.07	

## Data Availability

Publicly available datasets were analyzed in this study. These data are available at https://www.cdc.gov/nchs/nhanes/accessed February 20, 2024.
